# Use of virtual reality in children undergoing surgery

**DOI:** 10.3389/fped.2025.1633310

**Published:** 2025-07-16

**Authors:** Sabiha Bezgin, İrem Hüzmeli, Nihan Katayıfçı, Bilgi Asena Yıldırım, Ahmet Atıcı

**Affiliations:** ^1^ Department of Physiotherapy and Rehabilitation, Faculty of Health Sciences, Hatay Mustafa Kemal University, Hatay, Türkiye; ^2^ Department of Physiotherapy and Rehabilitation, Faculty of Health Sciences, Gazi University, Ankara, Türkiye; ^3^ Department of Physiotherapy and Rehabilitation, Institute of Health Sciences, Hatay Mustafa Kemal University, Hatay, Türkiye; ^4^ Department of Pediatric Surgery, School of Medicine, Hatay Mustafa Kemal University, Hatay, Türkiye

**Keywords:** child, general surgery, virtual reality, physical therapy, physiotherapy

## Abstract

**Objective:**

Early mobilization and exercise after surgery are very important to reduce the impact on respiratory system function. The aim of this study was to compare the effects of early mobilization with virtual reality and conventional physiotherapy methods on pulmonary function, dyspnea, exercise capacity, pain, and kinesiophobia in children undergoing surgery.

**Methods:**

Children aged 5–18 years who underwent various surgeries were randomly assigned to either a control group receiving conventional physiotherapy or a virtual reality group receiving additional virtual reality-based interventions. Interventions began on the day of surgery after anesthesia effects subsided and continued twice daily for two days. Outcome measures were performed before surgery and before discharge included pulmonary function tests, respiratory muscle strength, dyspnea, exercise capacity, dynamic balance, pain and kinesiophobia. Randomization was conducted using sealed-envelope drawing, and assessments were performed by a blinded physiotherapist.

**Results:**

A total of 27 children were included in the study, with 14 children in the control group and 13 children in the virtual reality group. When we analysed the results of pulmonary function tests, we observed that maximum inspiratory pressure increased in both groups, whereas maximum expiratory pressure increased only in the virtual reality group (*p* = .01). As a result, there was no difference in dyspnea, exercise capacity, dynamic balance, pain, and kinesiophobia in both groups (*p* > .05).

**Conclusion:**

In conclusion, we found that early physiotherapy was effective in the respiratory parameters of children undergoing surgery. Early mobilization with virtual reality improved the maximal expiratory pressure of children undergoing surgery. Thus, the virtual reality can serve as an alternative method to facilitate mobilization in children.

**Clinical Trial Registration:**

ClinicalTrials.gov, identifier (NCT06882382).

## 1 Introduction

Surgical interventions are performed in pediatric patients for a variety of indications. A number of factors have been identified as potential causes of postoperative pulmonary impairment. These factors encompass pre-, intra-, and postoperative elements such as obesity, impaired ventilation–perfusion ratio, duration of anesthesia, pain, immobility, abdominal distension, and restrictive bandages (1, 2). Postoperative pain has been demonstrated to impede effective coughing, thus contributing to impaired pulmonary function and the development of dyspnea (3). It is evident that respiratory muscles play a pivotal role in the respiratory process. Therefore, it can be hypothesized that anesthetic drugs may affect the function of these muscles and cause dyspnea. Dyspnea, or difficulty breathing, has been associated with both central and peripheral mechanisms, as well as chemoreceptor mechanisms. These factors have the capacity to influence mood and body responses, resulting in increased respiratory muscle activity. Research has demonstrated that physical activity exerts a substantial physiological influence on the respiratory system (4). In order to evaluate the response of respiratory muscles to increased ventilatory demand, it is necessary to assess the respiratory muscles and mechanics during exercise (5). Additionally, a fear of movement, otherwise termed kinesiophobia, has been identified as a risk factor for developing post-surgical pain and may impede recovery. The identification of individuals experiencing kinesiophobia during the surgical process holds potential value in optimizing rehabilitation and preventing fear of movement (6).

In patients who undergo anesthesia, atelectasis occurs in the lungs during the early postoperative period (7). Atelectasis develops as a result of diaphragm dysfunction, decreased activity of surfactant, and obstruction of the airways by secretions. Postoperative respiratory dysfunction is the most prevalent complication, with the potential to result in morbidity and mortality (8). The development of this complication is contingent on factors such as the nature of the surgery, the anesthetic agent utilized, and the pharmacologic agent administered (9). It is acknowledged that even in the absence of complications, there is a decline in exercise capacity following surgical operations (10). It is imperative to acknowledge the significance of chest physiotherapy and general physiotherapy applications in minimizing complications in the pre- and postoperative periods, irrespective of the primary cause of the surgical operation.

Approaches are adopted within play activities to ensure that the child accepts physical therapy and rehabilitation practices and to make them enjoyable. This approach has been demonstrated to promote active engagement among children and to cultivate their motivation (11). Among the methods employed in technological rehabilitation, virtual reality (VR) and game therapies are at the forefront (12). VR is a rehabilitation modality that enables patients to engage in exercise within a virtual environment, thereby facilitating the attainment of enduring outcomes. The employment of the VR method has been demonstrated to be both motivational and to facilitate appropriate movements for the purpose of the treatment, thus enabling its performance in a more functional manner (13). In addition to reducing pain, VR practices have been demonstrated to enhance pulmonary functions and increase functional capacity.

The objective of the present study was to compare the effects of VR physiotherapy and conventional physiotherapy on pulmonary function, dyspnea, exercise capacity, pain, and kinesiophobia during the early postoperative period in children.

## 2 Materials and methods

### 2.1 Study design

The current study was conducted between June 2022 and January 2023. Permission was obtained from the Hatay Mustafa Kemal University Clinical Research Ethics Committee (2022/29). The present trial was registered at ClinicalTrials.gov (Number NCT06882382). Written informed consent was obtained from all patients. All procedures were performed in accordance with the relevant guidelines and regulations, as well as the 1964 Helsinki Declaration and its subsequent revisions, or comparable ethical standards. The present trial has been reported in accordance with the CONSORT statement.

### 2.2 Sample size

The statistical analysis software SPSS 26.0 (Armonk, NY: IBM Corp.) was used, and the sample size was determined using G*Power software based on the effect size (ES = 1.8) from a previous study (14). A power analysis was conducted to determine the appropriate sample size for the study, considering a 90% power and a 95% confidence interval. This analysis indicated that including 14 participants in the study would be suitable.

### 2.3 Assessment parameters

#### 2.3.1 Pulmonary function test assessment

The pulmonary function test was measured using a portable spirometer. According to the American Thoracic Society/European Respiratory Society (ATS/ERS) criteria, the following parameters were measured: forced vital capacity (FVC), forced expiratory volume in the first second (FEV1), the ratio of forced expiratory volume in the first second to FVC (FEV1/FVC), peak expiratory flow rate (PEF), and forced mid-expiratory flow rate (FEF 25%–75%). The test was performed in the sitting position. The most optimal of at least three technically adequate manoeuvres that demonstrated 95% consensus amongst the participants was selected for statistical analysis. Pulmonary function test parameters were expressed as percentages of expected values according to age, height, body weight, and gender (15).

#### 2.3.2 Respiratory muscle strength assessment

The evaluation of respiratory muscle strength was conducted by means of an intraoral pressure measuring device. The maximal inspiratory pressure (MIP), inspiratory muscle strength, maximal expiratory pressure (MEP), and expiratory muscle strength were the focus of the evaluation. MIP was measured using the intraoral pressure measurement method. This process entailed the execution of rapid and profound inspiration within the residual volume following maximal expiration. MEP was recorded by performing rapid and deep expiration at total lung capacity after maximum inspiration. The children were encouraged to record the best performance verbally (16).

#### 2.3.3 Dyspnea assessment

The evaluation of dyspnea was conducted using the Modified Borg Scale (MBS). The scale, which was developed to measure the effort expended during physical exercise, consists of 10 items describing the severity of dyspnea according to its degree. According to the scale, the severity of dyspnea increases from 0 to 10 (17).

#### 2.3.4 Exercise capacity assessment

The 1-minute step test (1-ST) and the 10-meter walk test (10-MWT) were performed. In the 1-ST, children were instructed to step reciprocally up and down a 6-inch step for 1 min, and the number of steps taken was recorded (18). In the 10-MWT, children walked at a normal walking speed within a 10-meter section of a 14-meter walking distance. This was done to prevent the effects of the acceleration and deceleration phases of walking, and the time was recorded in seconds (19).

#### 2.3.5 Dynamic balance assessment

Participants' balance was assessed using the Timed Up and Go Test (TUG). The TUG is a clinical test that measures various components, including walking speed, postural control, functional mobility, and balance. The child was instructed to rise from a chair lacking armrests, proceed to a sign situated at a distance of 3 meters, and then assume a seated posture. The duration of the intervention was recorded in seconds (20).

#### 2.3.6 Pain assessment

The Numerical Pain Scale and Wong Baker Scale were employed to assess pain. The numerical pain scale is a tool used to assess numerical values between 0 and 10 points (0 = no pain, 10 = unbearable pain) (21). The scale was presented to the children, and its function was elucidated, after which they were invited to assign a numerical value to their pain. The Wong-Baker Scale is a method of evaluating pain intensity that utilizes six different facial expressions. The scale has been determined to be valid and reliable for the assessment of acute pain in school-age children. This conclusion has been reached due to the fact that children of this age are able to comprehend the scale with minimal explanation. The following evaluation of the scores obtained from the scale is proposed: The scale ranges from 0 to 10, with 0 representing no pain and 10 representing unbearable pain (22).

#### 2.3.7 Kinesiophobia assessment

Tampa Kinesiophobia Scale (TKS) was employed to evaluate the participants' levels of kinesiophobia. The scale comprises 17 questions designed to assess the individual's fear of movement, with a total score ranging from 17 to 68. A high score on the scale indicates a high level of kinesiophobia (23).

### 2.4 Randomization

Following the administration of screening and informed consent, the participants were randomly allocated into two equal groups. Participants were randomly assigned to study arms by drawing lots from a sealed container.

### 2.5 Blinding

The physiotherapist who was unaware of the group assignments performed the evaluations in a quiet environment in the physiotherapy room. It was not possible for the practitioners to be unaware of the study's nature due to the design of the study.

### 2.6 Interventions

The subjects of the study were divided into two groups, with the allocation to groups being made at random. All children participating in the study were mobilized in the early postoperative period. Subsequent to the dissipation of the effects of anesthesia, children were incorporated into physiotherapy practices. The day of the surgical procedure was designated as day 0, and the following days were designated as days 1 and 2. The children were discharged at the end of day 2. Physiotherapy interventions were initiated on the first day following the dissipation of the anesthetic effects, with sessions conducted twice daily. The control group underwent conventional physiotherapy interventions, encompassing normal joint movements, chest physiotherapy, and mobilization for a duration of 40 min each. Conversely, the experimental group received an additional 20 min of VR application per day, in conjunction with the conventional physiotherapy interventions. The technology of VR was realized through the utilization of the Microsoft Xbox Kinect 360 device. The Xbox Kinect 360 is a low-cost game console that facilitates interaction with the virtual environment through bodily movements, thereby obviating the need for gloves or a remote control during use. The system incorporates a pair of infrared depth sensors and a standard red, green, and blue camera. In conjunction with three-dimensional detection of a person's movements, this apparatus generates images that can be viewed on a television screen. The Xbox Kinect 360 utilizes a screen or television to which the sensor, adapter, controller, and equipment are connected. A curated selection of existing games was made based on a set of criteria that included entertainment value, musicality, visual appeal, and appropriateness for children. The selection of games was intended to promote children's involvement. The evidence indicates that the specified criteria have been satisfied, as demonstrated by the observation that River Rush, 20,000 Leaks, Rally Ball, Reflex Ridge, and Space Pop games were played.

### 2.7 Statistical analyses

The normality of the data was assessed using the Shapiro–Wilk test, and data that were normally distributed were presented as mean (standard deviation). Subsequently, an independent samples *t*-test was employed to compare the baseline characteristics of the groups, with the results expressed as mean differences and 95% confidence intervals. Nominal data were analyzed using the chi-square test. An intention-to-treat analysis was conducted, with all participants receiving either early mobilization or early mobilization plus VR as assigned. The data from all patients were then subjected to analysis based on their respective randomization assignments. The multiple imputation method was employed to address missing data and minimize the risk of underestimating variance. A series of analyses were conducted to evaluate the differences between the pre- and post-test conditions, as well as between the control group (CG) and the virtual reality group (VRG). The analyses employed included an analysis of covariance (ANCOVA), which was used to assess variations in respiratory muscle strength, pulmonary function, dyspnea, fatigue, exercise capacity, dynamic balance, pain, and kinesiophobia. The baseline measurements were utilized as covariates in the analyses. Subsequent *post hoc* comparisons for alterations in continuous variables within groups were manually adjusted using the Bonferroni test. A *p*-value of less than.05 was considered to be statistically significant.

## 3 Results

**Figure 1:**
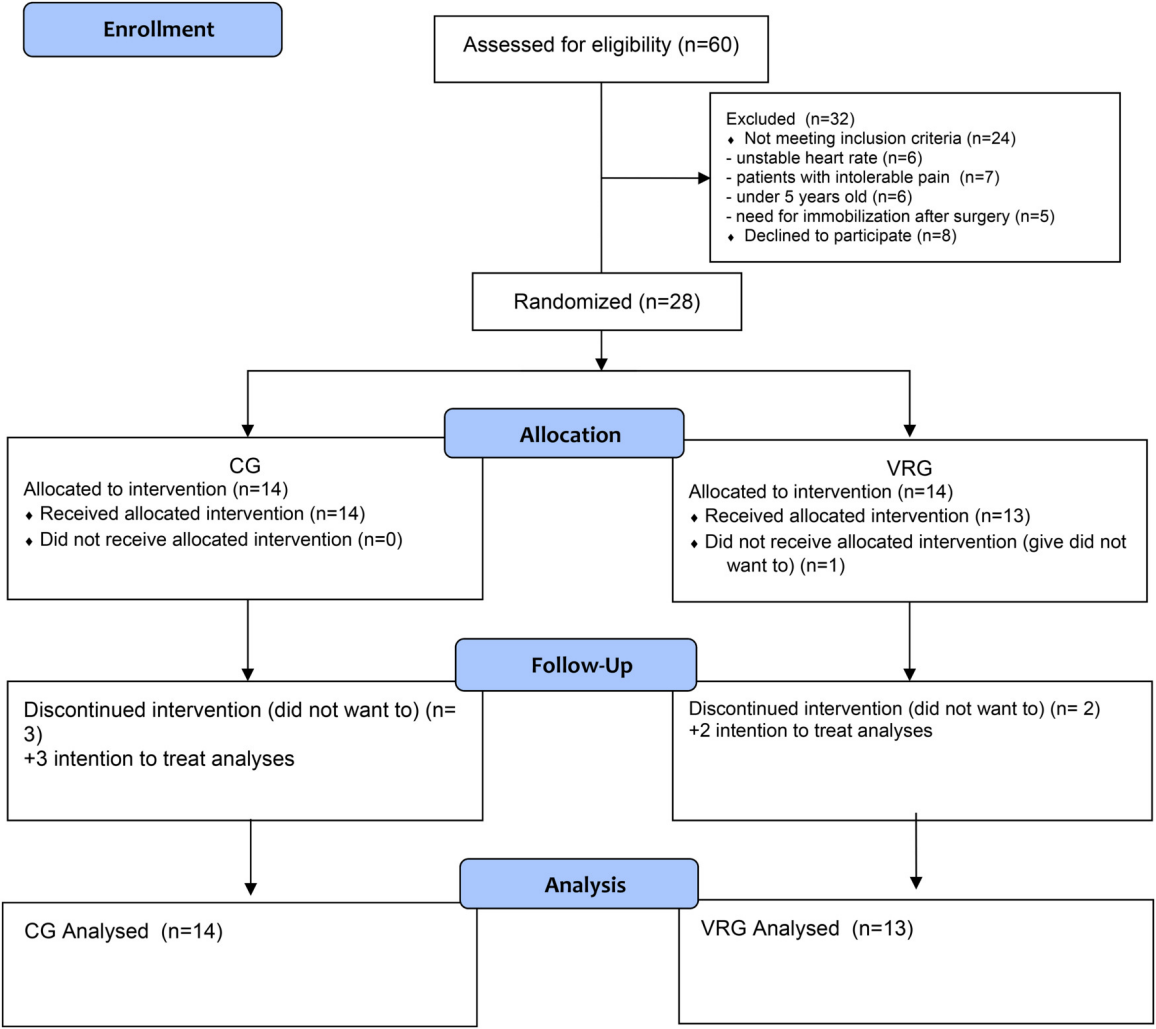
Flow chart of the study.

Sixty children hospitalized in the paediatric surgery were included in the study, and 32 were excluded twenty-seven children between the ages of 5 and 18 who had undergone surgery for inguinal hernia, undescended testicle, distal hypospadias, appendicitis, gallbladder, spleen and mass surgeries and who did not have visual and auditory sensory problems, were included in the study. Children who required immobilization after surgery and had a chronic disease that would affect pulmonary function were excluded from the study. Twenty-eight participants were randomly assigned to two groups: CG or VRG. Two people from the VRG group and three people from the CG group left the study because they did not want to participate (Figure 1).

### 3.1 Demographic and clinical characteristics

Participants' demographic and clinical characteristics were statistically similar and displayed in Table 1. The mean age was 11.57 ± 4.94 years in CG and 11.46 ± 5.17 years in VRG. More than half of the participants were male, and the proportion of those who underwent surgery due to masses was high in both groups (*p* > .05; Table 1).

**Table 1:** Demographic and clinical characteristics of the children.

Variables	CG (*n* = 14) X ± SD/Median (IQR)	VRG (*n* = 13) X ± SD/Median (IQR)	Mean difference [95%CI]	p
Age (years)	11.57 ± 4.94	11.46 ± 5.17	0.10 [−3.89 to 4.11]	0.995
Sex				0.440
Female, Male n/%	4/%28.6, 10/71.4	6/46.2, 7/53.8		
BMI (kg/m^2^)	18.36 ± 4.80	18.67 ± 3.25	−0.30 [−3.58–2.97]	0.848
Age of the mother (years)	39.57 ± 8.33	38.07 ± 6.68	1.4 [−4.48 to 7.47]	0.614
Education of the mother, n/%				
Literate	2/14.3	2/15.4		0.734
Primary school	4/28.6	3/23.1		
Secondary school	3/21.4	3/23.1		
High school	4/28.6	2/15.4		
Bachelor's degree	1/7.1	1/7.7		
Master's degree	0/0	2/15.4		
Surgery history, n/%				
Inguinal hernie	1/7.1	2/15.4		0.453
Distal hypospadias	1/7.1	1/7.7		
Undescended testicle	1/7.1	4/30.8		
Appendicitis	2/14.3	1/7.7		
Others	9/64.4	5/38.5		

BMI, body mass index; X, mean; SD, standart deviation; Chi-Square Test; independent samples test.

*p* < 0.05.

Table 2 presents the effectiveness of VR and early mobilization on postoperative children, focusing on outcomes such as respiratory muscle strength, pulmonary function, dyspnea, exercise capacity, dynamic balance, pain, and kinesiophobia.

**Table 2:** A comparison of the effects of virtual reality and early mobilization on postoperative children, focusing on outcomes such as respiratory muscle strength, lung function, dyspnea, dynamic balance, exercise capacity, pain, and kinesiophobia.

Variables	CG (X ± SD) *n* = 14			VRG (X ± SD) *n* = 13			Treatment effect p
	Post op day 0	Post op day 2	Within group p	Post op day 0	Post op day 2	Within group p	
MIP H_2_O	88.14 ± 20.56	94.07 ± 20.18	0.002*	75.69 ± 9.41	81.38 ± 10.52	0.015*	0.655
%MIP	94.74 ± 57.33	100.94 ± 60.35	0.422	96.17 ± 26.08	102.91 ± 26.77	0.406	0.377
MEP cmH_2_O	92.28 ± 18.29	90.82 ± 16.02	0.625	68.76 ± 9.16	77.61 ± 8.06	0.001*	0.054
% MEP	91.28 ± 35.01	90.31 ± 34.91	0.556	76.04 ± 37.97	85.15 ± 39.83	<0.001	0.001*
% FEV1/FVC	88.65 ± 11.68	86.28 ± 7.39	0.987	81.97 ± 9.02	84.69 ± 7.64	0.696	0.789
MBS (0–10)	0.75 ± 1.75	0.62 ± 1.18	0.395	0.80 ± 1.75	1.00 ± 1.63	0.562	0.523
1^ST^ (repitation)	32.35 ± 5.42	30.72 ± 3.64	0.723	26.15 ± 5.41	27.35 ± 4.16	0.383	0.415
10-MWT (sec)	7.50 ± 1.25	7.12 ± 2.19	0.342	7.49 ± 1.08	7.28 ± 1.11	0.628	0.752
RRrest (breathe/min)	22.87 ± 2.23	23.62 ± 3.20	0.995	26.70 ± 5.53	24.80 ± 6.42	0.494	0.692
TUG (sec)	6.69 ± 1.31	6.67 ± 1.00	0.145	6.54 ± 0.89	6.61 ± 0.98	0.150	0.791
*Δ*RR	1.57 ± 1.98	4.20 ± 5.36	0.676	4.40 ± 4.16	2.50 ± 3.50	0.693	0.582
Pain intensity (0–10)	1.18 ± 2.29	0.78 ± 1.76	0.583	2.07 ± 2.36	1.00 ± 1.95	0.051	0.081
Wong baker (0–10)	1.18 ± 2.46	0.71 ± 1.68	0.507	2.16 ± 2.16	1.09 ± 1.78	0.550	0.201
TKS score	22.14 ± 14.83	23.22 ± 12.87	0.922	29.66 ± 3.70	28.10 ± 3.08	0.774	0.787

CG, control group; VRG, virtual reality group; MIP, maximal inspiratory pressure; MEP, maximal expiratory pressure; FEV1/FVC, forced expiratory volume/forced Vital capacity; MBS, modified borg scale, 1st, 1 min step test; TUG, time up go test; 10-MWT, 10 min walking test; RR, respiratory rate; *Δ*, difference of post-pre results; TKS, tampa kinesiophobia scale; X, mean; SD, standart deviation.

*p* < 0.05, Ancova Test.

### 3.2 Pulmonary function and respiratory muscle strength

**Figure 2:**
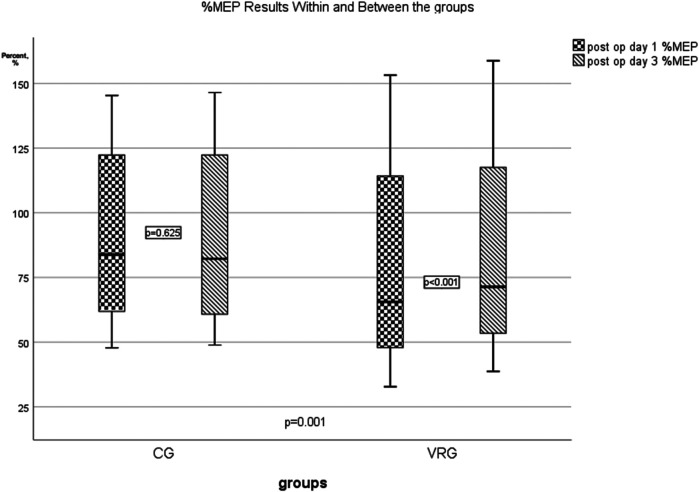
Treatment effect between the groups in % MEP results.

A statistically significant difference in MIP results was observed within both the CG (*p* = .002) and VRG (*p* = .015) between the pre- and postoperative periods, but no treatment effect was identified between the groups. Statistically significant improvements in MEP (*p* < .001) and %MEP (*p* = .001) were observed only within the VRG. A significant treatment effect was observed only for %MEP (*p* = .001, Figure 2) between the groups. FEV1/FVC% were similar between and within the groups (*p* > .05; Table 2).

### 3.3 Dyspnea, exercise capacity, dynamic balance, pain and kinesiophobia

Although improvements were noted within and between the groups for dyspnea, 10-MWT, 1-ST, TUG, respiratory rate, pain intensity and kinesiophobia, no statistically significant treatment effects were found (*p* > .05).

## 4 Discussion

The objective of the present study was to examine the effects of early mobilization with VR on postoperative pulmonary function, respiratory muscle strength, dyspnea, exercise capacity, dynamic balance, pain, and kinesiophobia in children undergoing surgery. The study demonstrated comparable enhancements in inspiratory muscle strength across the study groups. However, expiratory muscle strength exhibited a more pronounced improvement in the VRG group. Post-surgical assessments revealed the preservation of respiratory function, dyspnea, exercise capacity, dynamic balance, pain, and kinesiophobia in both groups. VR offers numerous possibilities for applications in healthcare and has been effectively utilized in adult healthcare environments, including in pediatric populations (24).

Microsoft Xbox 360 Kinect, a popular video game console, has demonstrated its efficacy as a rehabilitation and therapeutic tool by incorporating full-body movements in an engaging and enjoyable manner (25). The utilization of videogames in therapeutic applications has been documented in several studies. For instance, Basha et al. (26) reported the use of videogames in treating patients with acute burns. Similarly, Howcroft et al. (27) explored the use of videogames in the treatment of children with cerebral palsy. Additionally, Shin et al. (28) investigated the use of videogames in stroke rehabilitation. However, the present study is the first to compare the acute effects of early mobilization with VR on postoperative respiratory muscle strength, respiratory function, dyspnea, exercise capacity, dynamic balance, pain, and kinesiophobia in children. In the present study, inspiratory muscle strength demonstrated comparable improvements in both groups. However, expiratory muscle strength exhibited a more pronounced enhancement in the VRG with Xbox group following the second postoperative day. The observed immediate increase in inspiratory and expiratory muscle strength may be attributable to a reduction in pain experienced by children following surgery. Furthermore, the study found that children did not demonstrate respiratory muscle weakness either before or after surgery. Furthermore, the children demonstrated unremarkable respiratory function and did not manifest symptoms of dyspnea. Physiotherapy plays a crucial role both before and after surgery. A 30-min preoperative physiotherapy education session administered prior to surgery has been demonstrated to facilitate the maintenance of pulmonary function and functional capacity following open abdominal surgery (29, 30). A study demonstrated that forced vital capacity (FVC) exhibited enhancement five days following surgery for the preoperative physiotherapy education group in comparison to the postoperative physiotherapy group (30). In the present study, preoperative physiotherapy education was administered to both groups. Early mobilization has been demonstrated to facilitate the preservation of respiratory function following surgical intervention.

The acute effects on exercise capacity are not well understood. A study demonstrated that 12 weeks of training with Xbox was more effective than traditional exercises in improving exercise capacity in children with burns (26). Another study demonstrated an enhancement in 6-MWT distance on the fifth day following surgery, which approximated or equaled the preoperative levels after the preoperative physiotherapy education group and postoperative standard physiotherapy group (30). The present study demonstrated that postoperative surgical interventions did not result in a decline in exercise capacity among the study participants. The Xbox and mobilization groups both demonstrated the capacity to preserve exercise capacity during the acute phase. It was observed that dynamic balance was maintained in both the Xbox and mobilization groups. Furthermore, the 10-MWT time, which has been demonstrated to be a reliable predictor of falls risk, was maintained in both study groups. In the present study, the pre- and postoperative 10-MWT scores of all children were below the established cut-off score (>10 s) (31). The observed reduction in pain may have contributed to the preservation of balance (32).

Postoperative pain management has been demonstrated to play a pivotal role in the improvement of acute outcomes, reduction of hospital stays, and enhancement of patient satisfaction. Pain memory that develops early in childhood has been demonstrated to persist throughout life and exert a significant influence on quality of life (33, 34). Multimodal nonpharmacological strategies, comprising cognitive-behavioral, sensory-distraction, and physical techniques, have been shown to reduce both postoperative pain and kinesiophobia in children. For instance, the integration of therapeutic play, such as puppetry and medical clowns, with sensory distraction techniques, including music, video, and VR, has been demonstrated to reduce self-reported pain scores and fear of movement. This, in turn, enables earlier walking and participation in physiotherapy interventions (35). VR applications have been demonstrated to be an effective method for alleviating pain in children (36). However, the majority of these devices have been utilized for medical procedures, including venous access, dental care, oncological care, burn care, and VR. These devices function as a supplementary pain management method for hospitalized pediatric burn patients receiving physical therapy (37, 38).

It has been reported that children who experience postoperative pain remember more pain-related words, experience anxiety in adulthood, and try to avoid medical procedures, compared to their healthy peers. In these respects, the reduction in postoperative pain in the present study is a clinically significant result. Relaxation training (deep breathing, guided imagery) and massage therapy further weaken nociceptive signaling and muscle tension, reducing pain-related anxiety and subsequent movement avoidance. In our study, game-based approaches were used through VR. In our study, sensory (pain intensity) and emotional (fear of pain/movement) recovery dimensions were addressed with respiratory physiotherapy and virtual reality applications, and it was found that it encouraged early mobilization and improved lung function (35, 39). In addition, the kinesiophobia level was preserved after surgery. The reduction in pain and mobilization with the guidance of a physiotherapist may have prevented kinesiophobia. Therefore, it is essential to maintain early mobilization to prevent complications after surgery. The VR should be an alternative approach for children to provide mobilization.

Similar to our study, postoperative pain control has been shown to reduce patient pain, facilitate earlier mobilization and the ability to perform activities of daily living, and increase patient satisfaction (40). Along with pain reduction, targeted rehabilitation effects such as improvement of respiratory function, prevention of kinesiophobia, and preservation of muscle strength can be achieved with early mobilization.

Opioids are very important for pain management, but they have undesirable side effects. The need for complementary nonpharmacological interventions to improve pain control and reduce opioid dependence has been emphasized. As stated in a recently published study, the successful implementation of nonpharmacological pain management strategies largely depends on the education and support of healthcare professionals (41). Bezgin et al. (42) showed that video-based game application in children undergoing surgery has a positive effect on acute pain in children undergoing surgery. Suleiman-Martos et al. (43) emphasized that game-based strategies can improve the emotional health of patients and accelerate postoperative recovery as a result of their systematic review and meta-analysis investigating the effect of game-based approaches on preoperative pain in children. In the present study, pain levels declined, but this reduction did not reach statistical significance. In relation to this result of our study, it was shown that VR applications in the early postoperative period can contribute to pain management in a nonpharmacological way.

### 4.1 Limitations of the study

Our study is very valuable in terms of emphasizing the conclusion that VR technology can be used for early mobilization in children undergoing surgery. In addition, studies with a larger sample group are recommended. Furthermore, the comparison of children who underwent surgery with different diagnoses and the fact that respiratory functions were not evaluated immediately after surgery are considered limitations in our study.

## 5 Conclusions

In summary, this study demonstrated that physiotherapy combined with VR increased inspiratory muscle strength and improved expiratory muscle strength on the second day after surgery compared to conventional physiotherapy. Furthermore, both groups exhibited comparable preservation of respiratory function, dyspnea, exercise capacity, dynamic balance, pain, and kinesiophobia post-surgery. Physiotherapy is imperative both prior to and following surgery. Consequently, the virtual reality games can serve as an alternative modality to facilitate mobilization in children.

## Data Availability

The raw data supporting the conclusions of this article will be made available by the authors, without undue reservation.
